# Health economic evaluation of an internet intervention for depression (deprexis), a randomized controlled trial

**DOI:** 10.1186/s13561-020-00273-0

**Published:** 2020-06-16

**Authors:** Viola Gräfe, Steffen Moritz, Wolfgang Greiner

**Affiliations:** 1 Department of Health Economics and Health Care Management, School of Public Health, Bielefeld University, Universitätsstraße 25, 33615 Bielefeld, Germany; 2 Department of Psychiatry and Psychotherapy, University Medical Center Hamburg-Eppendorf, Martinistraße 52, 20246 Hamburg, Germany

**Keywords:** Economic issues, Outcome studies, Health economic evaluation, E-mental-health, Deprexis, Depression, Randomized controlled trial

## Abstract

**Background:**

Depressive disorders often remain undiagnosed or are treated inadequately. Online-based programs may reduce the present treatment gap for depressive disorders and reduce disease-related costs. This study aimed to examine the potential of the internet intervention “deprexis” to reduce the total costs of statutory health insurance. Changes in depression severity, health-related quality of life and impairment in functioning were also examined.

**Method:**

A total of 3805 participants with, at minimum, mild depressive symptoms were randomized to either a 12-week online intervention (deprexis) or a control condition. The primary outcome measure was statutory health insurance costs, estimated using health insurers’ administrative data. Secondary outcomes were: depression severity, health-related quality of life, and impairment in functioning; assessed on patient’s self-report at baseline, post-treatment, and three-months’ and nine-months’ follow-up.

**Results:**

In both groups, total costs of statutory health insurance decreased during the study period, but changes from baseline differed significantly. In the intervention group total costs decreased by 32% from 3139€ per year at baseline to 2119€ in the study year (vs. a mean reduction in total costs of 13% in the control group). In comparison to the control group, the intervention group also showed a significantly greater reduction in depression severity, and impairment in functioning and a significantly greater increase in health-related quality of life.

**Conclusion:**

The study underlines the potential of innovative internet intervention programs in treating depressive disorders. The results suggest that the use of deprexis over a period of 12 weeks leads to a significant improvement in symptoms with a simultaneous reduction in the costs of statutory health insurance.

## Introduction

Mental illnesses are responsible for a considerable part of the burden of disease and health care expenditure in Germany and other countries. They account for about 13% of the direct medical costs in Germany (thereof 19% due to depression) and cause considerable indirect costs [1]. The most common form of depressive disorders is major depression. The lifetime prevalence of a major depression is estimated at 11.6% to 13.0% in German adults, with women having nearly twice as high a risk of disease compared to men [2–6]. Aggravatingly, depressive episodes often persist for longer periods of time and become chronic [7]. From a societal perspective, depressive disorders are associated with a substantial loss of resources. Compared to people without depression, patients with depressive disorders report twice as many days of incapacity for work [8]; employees had an average absence of 51.8 days due to depressive episodes in 2014 [9]. In addition to indirect costs due to disease related productivity losses, depressive disorders are associated with high health care costs. Thus, the estimated annual direct treatment costs for Germany range between €686 [10] and €€2073 [11] per patient within different studies. The differences in average patient costs can be traced back to various conceptual issues, different methodological costing procedures and large differences in sample sizes. A recent study by Wagner et al. reports annual depression-related costs of €797, which is far closer to the €686 reported by Friemel et al. than to the €2073 reported by Salize et al., which seem rather overestimated [12, 13]. The total direct costs of depression in Germany were estimated at 5,2 billion Euro for the whole population [6, 14].

Despite differentiated guidelines and a well-developed health care system, depressive episodes are rarely identified early and treated adequately. Only one-third of all clearly clinically relevant depressive disorders are detected [15]. This globally documented treatment gap in the management of mental illnesses [16] may be counteracted by internet-based self-help interventions. This form of intervention is particularly relevant as a treatment for mild to moderate depression [17, 18]. Advantages are low threshold, local and temporal independence, reductions in waiting time for face-to-face treatment, empowerment and anonymity [18, 19].

Different studies along randomized controlled trials and some meta-analyses have provided evidence for the clinical effectiveness of online-based therapy programs for the treatment of depression (especially in the treatment of mild to moderate depressive symptoms). A meta-analysis by Karyotaki et al. found that self-guided internet-based behavioral therapy was significantly more effective with respect to depressive symptom severity and treatment response in comparison to control conditions with a small effect size, on average [20]. Furthermore, Cijpers and colleagues demonstrated that self-guided psychological treatment had a small but statistically significant effect on participants with elevated levels of depressive symptomatology [17].

While there is strong evidence for the effectiveness of web-based treatments for depression, effects on health care costs have been less well researched. Only a few health economic evaluations have been reported, most focusing on guided less on unguided or minimally-guided internet interventions. Whereas most studies indicated that guided web-based interventions have the potential to be cost-effective [21], health economic evaluations of self-guided treatment programs tend to classify these interventions as not cost-effective with respect to the direct costs of health services or productivity losses [22–24].

Against this background, the present study was designed to examine, whether the use of the unguided-guided cognitive behavioral internet intervention deprexis over a period of 12 weeks in addition to care as usual leads to a significant reduction in direct health care costs within 12 months of observation.

## Methods

### Trial design

This prospective, parallel-group, randomized controlled trial compared an online intervention for depression (deprexis) to a control condition. Using an a priori generated list with random numbers, participants were randomized equally (1:1) to either a 12-week internet intervention for depression or a control arrangement (received care as usual and a brochure with general information about depressive disorders). The trial was approved by the ethics committee of the general medical council Westfalen-Lippe and the WWU Münster (Germany), and registered at the German Clinical Trials Register (identifier: DRKS00003564).

### Participants

Participants were recruited from a large co-operating sickness fund between February 2010 and May 2014. All insured persons with a confirmed diagnosis of a mild (F32.0) or moderate (F32.1) depressive episode, according to the German version of the International Classification of Diseases, were invited to participate in the trial. To be included, participants had to be at least 18 years old, insured with the co-operating sickness fund for not less than 1 year, to suffer from at least mild depressive symptoms, defined by scores of > 4 on the Patient Health Questionnaire-9 (PHQ-9), had to have internet access, and had to be able to communicate in German. Participants with suicidality (PHQ-9: item 9 > 0) were excluded from the study prior to inclusion. Written informed consent of the study procedure, the aims of the trial and the benefits and risks of participation was obtained from all participants online prior to baseline assessment.

### Interventions

Following a ‘routine care’ research approach (pragmatic RCT), all participants in the trial were permitted to use any form of treatment, including psychotherapy and antidepressant medication. In addition to care as usual, participants of the intervention group received 12-week access to the internet intervention program deprexis. This program consists of ten modules covering a variety of therapeutic content based on cognitive-behavioral therapy techniques such as problem solving, psycho-education, interpersonal skills of mindfulness and acceptance, plus one introductory and one summary module. All modules are supported by illustrations, audio recordings, or short summary sheets. The program is interactive in nature by engaging its users in exercises and by continuously asking for responses within simulated dialogues in order to tailor subsequent content [25]. It is recommended that one to two sessions of around 30 min per week are undertaken, whereby the duration of use can vary individually [26]. The intervention can be used with or without guidance by a clinician. We used an unguided program version in this trial. A detailed description of the program is given by Meyer et al. [25].

Participants in the control group received care as usual as well as an additional digital brochure with general information on depressive disorders and services for people seeking (self-)help.

### Assessments

The primary outcome measure was the costs of statutory health insurance. Health care costs were estimated using health insurers’ administrative data. Cost categories included were medication costs, expenditures for inpatient hospital treatment and for rehabilitation as well as sickness benefits. All costs incurred were taken into account, not only those caused by depression. To ascertain changes in outcomes over time, health care costs were assessed for two time periods: 1 year pre enrollment to the trial and 1 year post enrollment.

The economic evaluation was conducted from a payer perspective according to the methods set out in the German recommendations on health economic evaluation [27]. Thus, indirect costs due to absenteeism or presenteeism, patients’ time and travelling costs, were excluded from the analysis. Program costs were also excluded from the analysis, as these are negotiated individually with clients such as health insurance companies and vary depending on usage circumstances [26]. Information on the amount of the fee is kept secret for competitive reasons and therefore not available for the German health care market. The costs of a single license for private persons (access to the program for 90 days after initial registration) amount to €297.50 including value-added tax [28]. Providing framework contracts with health insurance companies, the program-fees from the payer perspective can be assumed to be significantly lower than those for individuals.

Secondary outcomes were depression severity, health-related quality of life, and impairment in functioning. These outcomes were assessed retrospectively on patients’ self-report at baseline, post-treatment, three-months’ and nine-months follow-up using an online-based questionnaire.

Depression severity was measured, using the *Patient Health Questionnaire-9 (PHQ-9)*, a commonly used valid and reliable self-rating inventory for assessing depression diagnoses and monitoring depression severity [29, 30]. PHQ-9 consists of nine items, reflecting the criteria of depression in the Diagnostic and Statistical Manual of Mental Disorders (DSM-IV). Scores range from 0 to 27 points, with a score between 5 and 9 indicating mildly depressive symptoms and scores between 10 and 14 indicating moderate depression [30, 31].

Health-related quality (HRQoL) of life was assessed simultaneously utilizing the *Short Form Health Survey-12 (SF-12)* and the *EuroQol questionnaire (EQ-5D-3 L)*. Both instruments are widely used generic quality of life measures that have been applied in many different settings [32]. EQ-5D-3 L is a standardized instrument for describing and valuing health, consisting of a visual analog scale and a descriptive system which defines health across five dimensions (e.g. mobility, self-care or anxiety/depression), with each dimension specifying three levels of severity. By applying preference-based weights, each health state can be converted into a single summary index. Within this trial only the descriptive system was applied [32].

The SF-12 questionnaire is a reliable and sensitive instrument for measuring HRQoL in people with mental illness, consisting of 12 questions assessing the presence and severity of different aspects of functioning and limitations due to emotional or physical health problems. It can be reported as a physical or a mental component summary scale [33–35]. Due to its good to excellent internal consistency and convergent validity it is comparable to its longer version, the SF-36, and is therefore the instrument of choice in longitudinal studies [36].

The *Work and Social Adjustment Scale (WSAS)* is a simple and short measure of self-reported functional impairment. Psychometric properties, validity and sensitivity to change of the five item-scale have been documented in several studies, including those focusing on the treatment of depression and mental distress [37, 38]. Participants with a score below 10 can be classified as unimpaired in functioning, a score between 10 and 20 is associated with a significant functional impairment and a score above 20 suggests a moderately severe or worse psychopathology [37].

### Sample size

The sample size calculation was based on an expected difference between the intervention and the control group on the main outcome variable “total costs of statutory health insurance” 12 months after enrollment on the trial. Based on an estimated reduction in total health care expenditure of 20%, a power of 0.80, an alpha level of 0.05 and a drop-out rate of 30%, 1750 participants were needed in each condition. The effect-size calculation was based on an analysis of claims data from the co-operating sickness fund for 2008 and 2009, considering costs for inpatient hospital treatment, outpatient medical care, outpatient paramedical services, rehabilitation, medication costs and sickness benefits.

### Data analysis

To assess the comparability between the study groups at baseline we calculated measures of central tendency and measures of variability. To determine the precision of mean values, 95%-confidence intervals were calculated. Chi-square tests and ANOVAs with Tukey’s post hoc test were applied for further examination of group differences.

To check whether the intervention also had an influence on costs independently from baseline costs, we conducted a difference in differences analysis. Hence, the difference in costs between baseline and the study period was calculated. The changes in mean costs were then examined for differences between study groups, using t-tests for independent samples (two-tailed). All costs are presented as mean, 5% trimmed mean, and 95%-CI of the mean for the year previous to study enrollment and for the study year. Furthermore, corresponding *p*-values are presented.

To describe the assessed secondary outcomes over time and to check the observed values for regularities, time series analyses were conducted. Comparative subgroup-analyses were applied for each of the secondary outcomes using a two-factor mixed-design ANOVA with observed means of the secondary outcomes as the within-subjects factor and the study group as between-subject-factor. To correct for violations of sphericity, the Greenhouse-Geisser adjustment was used when appropriate.

Effect sizes for the secondary outcomes are presented as Cohen’s *d*, which was calculated as difference between means of intervention and control group, divided by the pooled standard deviation of both groups. Following current standards, all effect sizes were calculated from the observed means of the study groups and defined as small (*d* = 0.2), medium (*d* = 0.5) and large (*d* = 0.8) [39, 40].

The statistical analyses were based on all observed data. We did not impute missing values as the statistical methods utilized were robust and valid for missing-at-random data and complete case analysis remains a very common case of handling missing data [41].

We performed our statistical analyses using IBM SPSS Statistics for Windows version 23.0. Review and preparation of claims data was carried out with Microsoft Excel 2016. The final cost variables were then reimported to IBM SPSS-statistics for further analyses.

## Results

### Participant flow and baseline characteristics

**Fig. 1:**
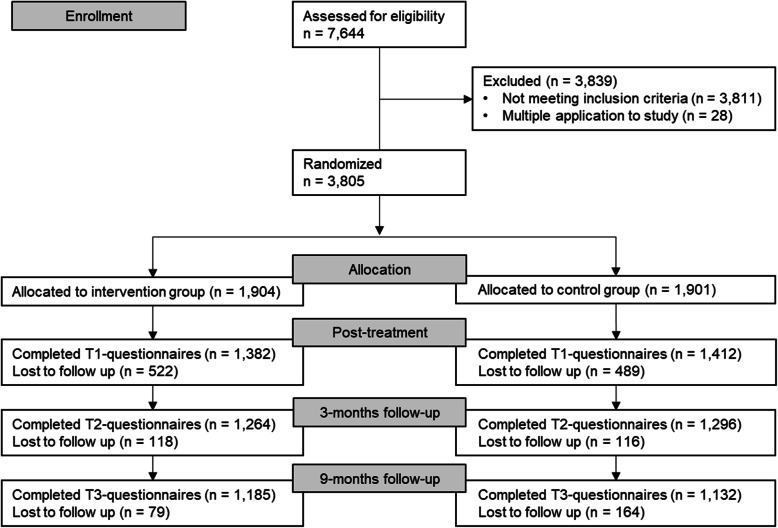
CONSORT participant flow diagram

As shown in Fig. 1, a total of 7644 applicants signed up for the study and were screened for inclusion and exclusion criteria. 3811 did not meet the inclusion criteria and thus had to be excluded from the trial. Most common exclusion criteria were the presence of suicidal feelings (53.19%; *n* = 2027), no insurance affiliation at the co-operating sickness fund (24.5%; *n* = 933) and a PHQ-9 score > 5 (11.2%; *n* = 426). Later, 28 participants were excluded from the analyses, because they gained multiple access to the study by applying several times using different pseudonyms. Finally, 3805 participants were randomized to either intervention (*n* = 1904) or control (*n* = 1901). The last 9-months follow-up assessment was performed in May 2014, by which time 62.24% (*n* = 1185) of the intervention group and 59.54% (*n* = 1132) of the control group had completed all questionnaires. No significant differences in rates of attrition were found between the study groups at post-treatment, three-months or nine-months follow-up. Neither randomization group, nor baseline costs, sex, age, educational status or family status were significantly associated with dropout status. Full information on participant flow is shown on the CONSORT flow chart (Fig. 1).

Participants in the intervention group did not significantly differ from those in the control group at baseline on any of the demographic variables or treatment history, indicating that randomization had been well balanced (see Table 1). Briefly, the modal participant was 46 years old, female, had completed middle secondary education (10 years of school, until age 16/17), was employed full-time, suffered from moderate self-reported depressive symptoms (PHQ-9: 12), and reported being in treatment for depression (especially drug therapy).

**Table 1:** Baseline sociodemographic clinical characteristics*

Characteristic	Intervention Group (*N*= 1,904)		Control Group (*N*= 1,901)		Total (*N*= 3,805)	
	**Mean**	**SD**	**Mean**	**SD**	**Mean**	**SD**
Age	45	11.0	46	10.7	45	10.9
Weekly working hours	33	16.0	34	15.9	33	15.9
	**N**	**%**^**a**^	**N**	**%**^**a**^	**N**	**%**^**a**^
Female	1496	79.2%	1491	78.8%	2987	79.0%
Nationality						
German	1819	97.0%	1823	97.1%	3642	97.0%
Other	57	3.0%	55	2.9%	112	3.0%
Highest academic qualification^b^						
Lower secondary	237	12.6%	213	11.4%	450	11.8%
Middle secondary	748	39.9%	759	41.6%	1507	40.2%
Higher secondary	547	29.2%	549	28.9%	1096	29.2%
University degree	335	17.9%	328	17.6%	663	18.4%
None of them, haven't got a graduation (yet)	4	0.2%	1	0.1%	5	0.1%
No graduation/ leaving certificate	4	0.2%	8	0.4%	12	0.3%
Vocational education (multiple responses possible)						
Apprenticeship	1136	44.7%	1125	44.4%	2261	44.6%
Vocational school	382	15.0%	376	14.9%	758	15.0%
Technical college	307	12.1%	291	11.5%	598	11.8%
University	425	16.7%	419	16.6%	844	16.6%
No degree	95	3.7%	110	4.3%	205	4.0%
Other degree	194	7.6%	210	8.3%	404	8.0%
Working status						
Self-employed	76	4.1%	91	4.9%	167	4.5%
Employee (part-time)	494	26.6%	513	27.6%	1007	27.1%
Employee (full-time)	698	37.6%	715	38.4%	1413	38.0%
Seeking work	235	12.7%	207	11.1%	442	11.9%
Student	111	6.0%	95	5.1%	206	5.5%
Pensioner	211	11.4%	205	11.0%	416	11.2%
Temporary employee	30	1.6%	34	1.8%	64	1.7%
Currently in treatment for depression	1036	55.2%	983	52.3%	2019	53.8%
Outpatient psychotherapy						
Currently	521	32.5%	495	31.1%	1016	31.8%
Recently	413	25.8%	455	28.6%	868	27.2%
Some time ago	667	41.7%	643	40.4%	1310	41.0%
Day-clinic treatment						
Currently	25	10.3%	18	7.4%	43	8.8%
Recently	56	23.0%	58	23.9%	114	23.5%
Some time ago	162	66.7%	167	68.7%	329	67.7%
Drug therapie						
Currently	871	53.8%	842	52.7%	1713	53.2%
Recently	338	20.9%	378	23.6%	716	22.3%
Some time ago	409	25.3%	379	23.7%	788	24.5%
In-patient stay in a psychiatric or psychosomatic clinic						
Currently	36	4.9%	29	4.0%	65	4.5%
Recently	190	26.0%	190	26.3%	380	26.2%
Some time ago	504	69.0%	504	69.7%	1008	69.4%

^a^valid percentage (excluding missing values)

^b^Highest academic qualification according to the German classification “Hauptschule” (“lower”, 9 years, until age 15/16), “Realschule” (“middle”, 10 years, until age 16/17), “(Fach)Hochschulreife/Abitur” (“highest”, 12 or 13 years, until age 17-19)

**Table 2:** Health care expenditures (in €) by sector and study condition

	Intervention (*n*=1,904)				Control (*n*=1,901)				
	Mean	5% trimmed mean	95% - CI of the mean	*p*-value within-group differences	Mean	5% trimmed mean	95% - CI of the mean	*p*-value within-group differences	*p*-value between-group differences^c^
**Total amount**^**a b**^									
Previous year	3,142.57	1,870.65	2,819.92 - 3,465.22	<0.001	3,130.93	1,892.93	2,781.83 - 3,480.03	0.044	0.041
Study year	2,121.91	1,130.73	1,876.43 - 2,367.39		2,694.57	1,438.86	2,383.38 - 3,005.77		
**Medication costs**									
Previous year	845.35	354.32	684.59 - 1,006.11	<0.001	747.02	332.51	623.29 - 870.75	0.002	0.140
Study year	524.16	260.34	434.45 - 613.87		567.31	259.74	467.64 - 666.97		
**Inpatient hospital treatment**									
Previous year	1,249.26	557.17	1,081.62 - 1,416.89	0.084	1,373.94	574.81	1,139.87 - 1,608.02	0.438	0.255
Study year	1,067.10	458.29	916.87 - 1,217.33		1,398.38	584.86	1,191.74 - 1,605.02		
**Rehabilitation**									
Previous year	23.63	0.00	11.07 - 36.19	0.926	33.67	0.00	11.37 - 55.96	0.869	0.549
Study year	24.55	0.00	9.95 - 39.15		45.40	0.00	26.01 - 64.79		
**Sickness benefit**									
Previous year	1,024.33	252.10	845.39 - 1,203.26	<0.001	976.30	222.86	808.39 - 1,144.19	0.012	0.162
Study year	506.10	14.37	380.18 - 632.02		683.48	69.93	529.62 - 837.33		

^a^exclusive outpatient costs

^b^Fees for the intervention were not included in the analysis, because they are negotiated individually with clients such as health insurance companies. The costs of a single licence for private persons amount to €297.50. Providing framework contracts with health insurance companies, the program-fees from payer perspective can be assumed to be lower than those for individuals

^c^refers to the estimated mean difference between both time-periods

### Health care expenditures

There were no significant differences in direct health care costs between the study conditions at baseline. During the study period total costs of statutory health insurance decreased in both groups, but changes from baseline differed significantly between the groups (t_df = 3803_ = 2.05; *P* = .04; see Table 2). While total costs decreased by 32% from €3143 per year at baseline to €2122 in the study year in the intervention group (t_df = 1903_ = 5.47; *P* < .001), these costs decreased by 13% in the control group (from €3131 to €2695; t_df = 1900_ = 2.02; *P* = .04). The significant difference in total expenditure changes could mainly be attributed to a bigger decrease of sickness benefits in the intervention group (intervention: - €518 vs. control: - €293), and an opposite trend in the development of costs for inpatient hospital treatment. Whereas mean costs for inpatient treatments decreased in the intervention group by €182, they increased slightly in the control group (+€24).

However, on closer examination of sector-specific health care costs, the internet intervention did not have a significant effect on changes in single cost-categories. Medication costs and expenditures for sickness benefits decreased significantly within both study groups, but changes did not significantly differ between groups – neither for medication costs (t_df = 3803_ = 1.48; *P* = .14), nor for sickness benefits (t_df = 3803_ = 1.40; *P* = .16), costs of inpatient hospital treatment (t_df = 3803_ = 1.14; *P* = .25) or rehabilitation t_df = 3803_ = 0.60; *P* = .54).

### Psychopathology and functional impairment

Based on mixed-design ANOVAs of the intention to treat sample, the intervention had a significant effect on depression severity, functional impairment, and HRQoL (whether assessed with the SF-12 mental summary scale, or with EQ-5D-3 L). In comparison to the control group, the intervention group showed a significantly greater reduction in PHQ-9 (F_2.81, 5602.08_ = 41.7; *P* < .001), a significantly greater decrease of impairment in functioning (F_2.77, 5518.60_ = 18.64; *P* < .001) and a significantly greater increase in HRQoL when assessed on the SF-12 mental health summary scale (F_2.92, 5819.66_ = 26.34; *P* < .001) and on EQ-5D-3 L (F_2.97,6115.28_ = 4.97; *P* = .002).

**Fig. 2:**
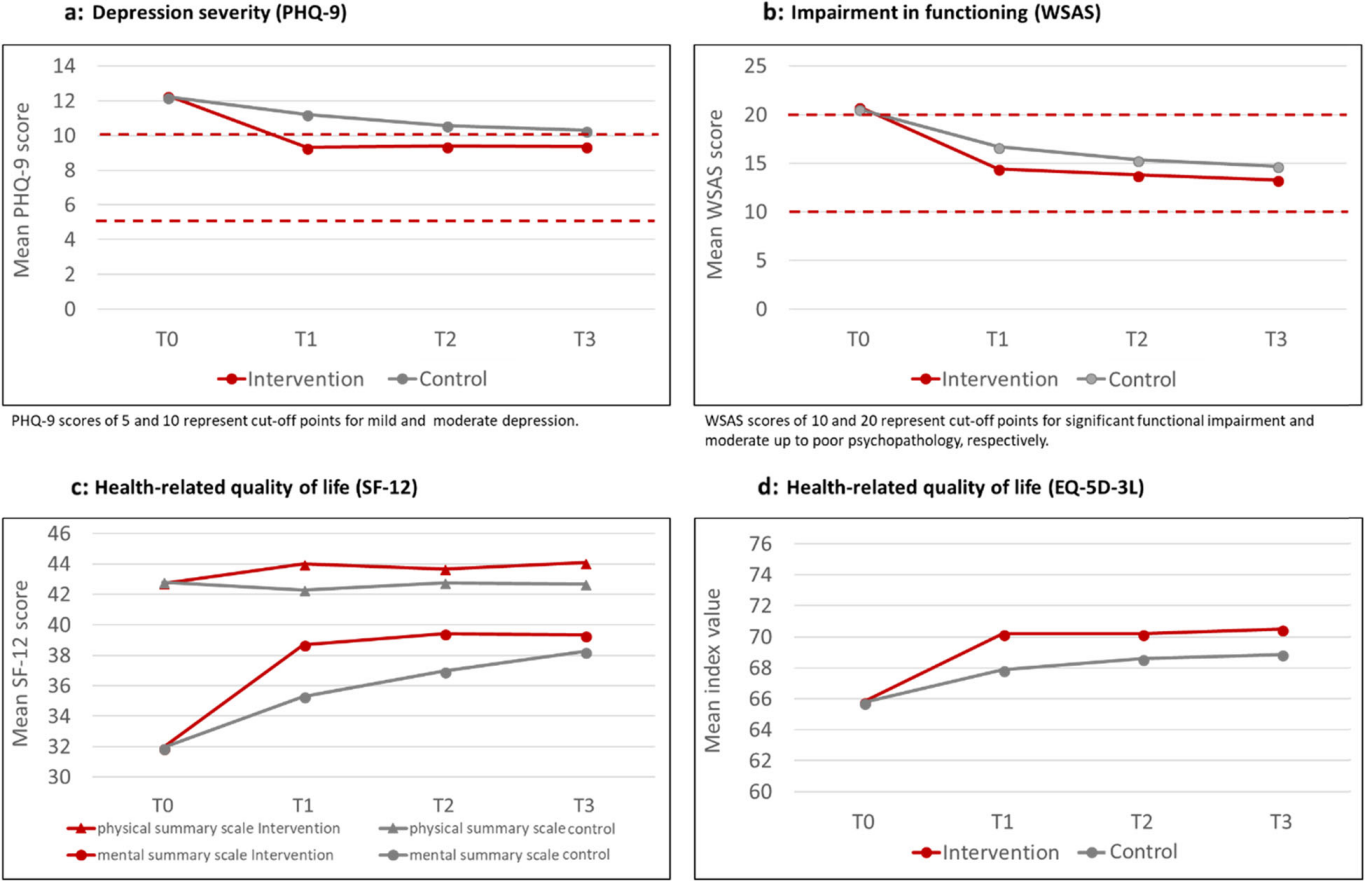
Secondary outcome measures by study condition and time

Across all secondary outcomes the intervention group showed a significantly greater improvement in measured effects at post-treatment assessment than the control group. While effects on the self-rating tools were relatively stable at follow-ups within the intervention group, the values of the control group were slowly approaching those of the intervention group. For detailed information on changes of secondary outcome measures see Fig. 2.

Even though the interaction between time and treatment group reached significance for all secondary outcomes, the between-group effect sizes differed from the small to medium range. Effect sizes for PHQ-9 and the SF-12 mental summary scale were larger than those for the other measures with *d* = 0.37 for PHQ-9 and *d* = 0.33 for SF-12 at post-assessment and analogously *d* = 0.23 and *d* = 0.22 at three-months’ follow-up. For SF-12 physical summary scale, EQ-5D-3 L and WSAS only small effect sizes could be determined (see Table 3 and Additional file  1: Table S1 (online supplementary)).

**Table 3:** Between-group effect sizes of secondary outcomes

	Post-assessment		3-monts follow-up		9-monts follow-up	
	Cohen's d	95% - CI	Cohen's d	95% - CI	Cohen's d	95% - CI
PHQ-9	0.37	0.29 - 0.44	0.23	0.15 - 0.31	0.15	0.07 - 0.23
SF-12 physical summary scale	0.18	0.10 - 0.25	0.09	0.01 - 0.17	0.14	0.05 - 0.22
SF-12 mental summary scale	0.33	0.25 - 0.40	0.22	0.15 - 0.30	0.09	0.01 - 0.18
EQ-5D-3L	0.14	0.07 - 0.22	0.10	0.02 - 0.17	0.09	0.01 - 0.18
WSAS	0.23	0.15 - 0.31	0.15	0.07 - 0.23	0.120.12	0.02 - 0.24

Cohen's *d* was calculated as the difference between the mean of the intervention and control group, divided by the pooled standard deviation of both groups. The effect sizes are defined as small (d = 0.2), medium (d = 0.5) and large (d = 0.8)

## Discussion

### Main results

This randomized controlled trial evaluated the potential of an innovative internet intervention program to reduce health care costs within 1 year of after starting the program use. The trial showed that the internet intervention deprexis had a significant effect on the direct costs of health care-utilization, and on measures of depression severity, HRQoL and functional impairment.

During the observation period the total costs of statutory health insurance decreased in both study groups, but changes from baseline did significantly differ between groups. While costs decreased by 32% (€1021) in the intervention group, costs decreased by 13% (€436) in the control group. As mentioned above, program costs were excluded from the analysis, as no price information is available for the German healthcare market (see 2.4). Additional scenario analyses have shown that the difference in total health care costs remains significant up to an amount of €34 per patient. If the fee exceeds an amount of €34, there would be no significant difference in total costs of statutory health insurance between intervention group and control group.

In addition to the effects on costs, the intervention was also showed to be effective in reducing disease-related secondary symptoms. In comparison to the control group, the intervention group gained from a significantly greater improvement of depressive symptoms, a significantly greater decrease of impairment in functioning a significantly greater increase in HRQoL. It is not conclusively proven whether the measured changes in secondary outcomes also represent a minimally important difference (MID). To the best of our knowledge, there is no secured evidence on the MID of the used instruments specifically for patients with depression. As the changes on PHQ-9 and WSAS reached the instruments defined cut-off-points within the intervention group, it can be assumed, that the changes in depression severity (measured with the PHQ-9) and in impairment in functioning seem to be clinically relevant.

In summary, the results on cost differences and effects point in the same direction and do not lead to different conclusions, indicating that the findings of our study are robust.

### Strengths and limitations

Some limitations should be considered when interpreting the trial results. First, the effect of the online therapy deprexis in gaining savings in health care costs from the payer perspective may have been underestimated, since outpatient health care cost were not available for the analysis. In particular, mild to moderate depressive disorders are commonly treated within the outpatient health care sector. Since the results of this study demonstrated significant differences in the change of total health care costs between the intervention and control group even though outpatient treatment costs were not available for the analyses, it can be assumed that the inclusion of outpatient treatment costs would reinforce the results. Recently published results from another randomized controlled trial on deprexis confirm this assumption. The study by Gräfe et al. suggest that the use of deprexis in combination with care as usual leads to a significant decrease in outpatient treatment costs, especially in those related to different types of psychotherapeutic treatment [42].

In addition to the outpatient treatment costs, the intervention costs could also not be included to the analysis. As described in the method section, program costs are negotiated individually with clients such as health insurance companies and vary depending on usage circumstances. Depending on the license-fees, the statistically significant difference in mean total costs at 12 months post-enrollment could be offset (see also section “main results”).

Another limitation exists with respect to the relatively high attrition rate at nine-months follow up. Only around half of those who had completed the baseline questionnaire and were enrolled to the study also completed the last follow-up questionnaire. Nevertheless, the study results can be assumed to be robust as neither randomization group, nor baseline costs, sex, age, educational status or family status were significantly associated with dropout status. Attrition rates at post treatment and at three-months follow-up are in line with previous trials of this intervention [43–46].

A final limitation that should be noted is the restricted transferability in terms of sociodemographic aspects. In comparison to the corresponding German general population, participants in our study had a higher educational level and women were overrepresented in the study. These findings are in line with previously published studies [47]. Thus, the higher proportion of women can be explained by a higher prevalence of depression in females. Furthermore, women are more likely to seek help than men. The higher educational level of participants within this trial could be explained by a higher demand for internet interventions by such people, which was shown for users of a web-based computer-tailored intervention promoting heart-healthy behaviors [48].

Along with the limitations mentioned above, our study also benefits from some important strengths. First, this trial used health insurers’ administrative data to estimate direct health care costs. The majority of currently published studies evaluating different e-mental health interventions and calculating their cost-effectiveness have been based on patients’ self-reports. Even though patient self-report questionnaires are a common and approved method to obtain costing data, they suffer from limitations due to recall bias, especially if recall-periods are long. In consequence, results may have been distorted by over- or underreporting [49]. As different studies have demonstrated, administrative data and self-report data provide different estimates of health-related resource-use, and of resulting costs. Particularly among people with mental disorders the discrepancy can be large [50, 51]. Since health-insurers’ administrative data are not biased due to memory failure and are based on expenses incurred, high reliability of results can be assumed.

In comparison to other recently published studies, this study also benefits from the large number of participants enrolled in the trial. To our knowledge, the present health economic evaluation is the largest published study, which was conducted alongside a randomized controlled trial focusing on costs and effects web-based treatment for depression [21]. Furthermore, our study profits from being specially powered to detect differences in costs. Hence, the power calculation for this trial was therefore based on expected savings in health care cost from the payer perspective, and not on an expected clinical outcome as in most other studies within this context.

## Conclusion

This study underlines the potential of innovative e-mental-health programs in treating depressive disorders. The results suggest that the use of deprexis over a period of 12 weeks in comparison to care as usual leads to a significant reduction in costs of statutory health insurance with a simultaneous reduction of depressive symptoms, an increase in health-related quality of life and a decrease of impairment in functioning. From a health-economic perspective, the use of the program can be recommended, as cost-savings from the payer perspective are in line with the clinical benefits gained.

## Supplementary information

**Additional file 1: Table S1.** Secondary outcomes by study condition and time.

## Data Availability

The data that support the findings are not publicly available, as the publication of the collected primary data is not covered by the informed consent.

## Abbreviations

DSM-IV: Diagnostic and Statistical Manual of Mental Disorders
EQ-5D-3 L: EuroQol questionnaire
HRQoL: Health-related quality
PHQ: Patient Health Questionnaire 9 Items
RCT: Randomized controlled trial
SF-12: Short Form Health Survey-12
