# Correction to: Health economic evaluation of an internet intervention for depression (deprexis), a randomized controlled trial

**DOI:** 10.1186/s13561-020-00280-1

**Published:** 2020-07-30

**Authors:** Viola Gräfe, Steffen Moritz, Wolfgang Greiner

**Affiliations:** 1 Department of Health Economics and Health Care Management, School of Public Health, Bielefeld University, Universitätsstraße 25, 33615 Bielefeld, Germany; 2 Department of Psychiatry and Psychotherapy, University Medical Center Hamburg-Eppendorf, Martinistraße 52, 20246 Hamburg, Germany

**Correction to: Health Econ Rev 10, 19 (2020)**

**https://doi.org/10.1186/s13561-020-00273-0**

Following publication of the original article [1], the authors reported an error in their paper after publication that the supplementary table was captured as Table 3 while Table 3 was used as the supplementary file. In addition the table headings were incorrectly assigned. The correct Table 3 is shown below. The original article [1] has been updated.

**Table 3:** Between-group effect sizes of secondary outcomes

	Post-assessment		3-monts follow-up		9-monts follow-up	
	Cohen’s d	95% - CI	Cohen’s d	95% - CI	Cohen’s d	95% - CI
PHQ-9	0.37	0.29–0.44	0.23	0.15–0.31	0.15	0.07–0.23
SF-12 physical summary scale	0.18	0.10–0.25	0.09	0.01–0.17	0.14	0.05–0.22
SF-12 mental summary scale	0.33	0.25–0.40	0.22	0.15–0.30	0.09	0.01–0.18
EQ-5D-3 L	0.14	0.07–0.22	0.10	0.02–0.17	0.09	0.01–0.18
WSAS	0.23	0.15–0.31	0.15	0.07–0.23	0.12	0.02–0.24

Cohen’s *d* was calculated as the difference between the mean of the intervention and control group, divided by the pooled standard deviation of both groups. The effect sizes are defined as small (d = 0.2), medium (d = 0.5) and large (d = 0.8)
