# Supplementary material for: Health economic evaluation of an internet intervention for depression (deprexis), a randomized controlled trial
Source: Health Econ Rev. 2020 Jun 16;10:19. doi: 10.1186/s13561-020-00273-0 (PMC7298860; doi:10.1186/s13561-020-00273-0)
Supplement: Supplementary file 1 — Additional file 1: Table S1. Secondary outcomes by study condition and time. [file 13561_2020_273_MOESM1_ESM.pdf]

	Intervention			Control		
	Mean	95% - CI of the mean	p-value within-group differences	Mean	95% - CI of the mean	p-value within-group differences
**PHQ-9**						
T0	12.38	12.19 - 12.57	T0 to T1: <0.001	12.28	12.09 - 12.48	T0 to T1: <0.000
T1	9.31	9.04 - 9.57	T1 to T2: 0.135	11.18	10.91 - 11.47	T1 to T2: <0.000
T2	9.37	9.07 - 9.66	T2 to T3: 0.464	10.60	10.30 - 10.90	T2 to T3: 0.008
T3	9.38	9.05 - 9.69	T0 to T3: <0.001	10.23	9.90 - 10.56	T0 to T3: <0.000
**WSAS**						
T0	21.04	20.67 - 21.43	T0 to T1: <0.001	20.74	20.36 - 21.12	T0 to T1: <0.000
T1	14.46	13.94 - 14.97	T1 to T2: 0.013	16.71	16.20 - 17.22	T1 to T2: <0.000
T2	13.88	13.33 - 14.45	T2 to T3: 0.289	15.38	14.81 - 15.94	T2 to T3: 0.003
T3	13.36	12.76 - 13.95	T0 to T3: <0.001	14.62	14.01 - 15.24	T0 to T3: <0.000
**SF-12 pysical summary scale**						
T0	42.72	42.24 - 43.21	T0 to T1: <0.001	42.80	42.34 - 43.26	T0 to T1: 0.211
T1	44.00	43.48 - 44.52	T1 to T2: 0.289	42.29	41.79 - 42.79	T1 to T2: 0.421
T2	43.66	43.09 - 44.23	T2 to T3: 0.336	42.76	42.22 - 43.30	T2 to T3: 0.919
T3	44.09	43.49 - 44.66	T0 to T3: <0.001	42.69	42.08 - 43.29	T0 to T3: 0.299
**SF-12 mental summary scale**						
T0	31.92	31.53 - 32.31	T0 to T1: <0.001	31.29	31.55 - 32.29	T0 to T1: <0.001
T1	38.27	38.14 - 39.30	T1 to T2: 0.280	35.30	34.79 - 35.82	T1 to T2: <0.001
T2	39.44	38.82 - 40.07	T2 to T3: 0.558	36.97	36.37 - 37.58	T2 to T3: 0.008
T3	39.34	38.69 - 40.01	T0 to T3: <0.001	38.26	37.59 - 38.93	T0 to T3: <0.001
**EQ-5D_3L**						
T0	65.78	65.09 - 66.48	T0 to T1: <0.001	65.75	65.06 - 66.45	T0 to T1: <0.001
T1	70.23	69.37 - 71.09	T1 to T2: 0.963	67.87	67.00 - 68.75	T1 to T2: 0.085
T2	70.21	69.28 - 71.14	T2 to T3: 0.907	68.60	67.67 - 69.52	T2 to T3: 0.648
T3	70.50	69.53 - 71.47	T0 to T3: <0.001	68.88	67.85 - 69.91	T0 to T3: <0.001
